# Proteome-wide QTL mapping enables gene-protein-phenotype metabolic network construction in a genetically diverse MASLD mouse model

**DOI:** 10.64898/2026.07.26.740836

**Published:** 2026-07-28

**Authors:** Margaret Lea Robinson, Wenyu Liu, Giorgia Benegiamo, Miller T. Williams, Gordon I. Smith, Samuel Klein, Johan Auwerx, Joshua J Coon

## Abstract

The limited treatment options available for the estimated 38% of adults worldwide affected by Metabolic Dysfunction Associated Steatotic Liver Disease (MASLD) are largely due to an incomplete understanding of the complex molecular networks underlying disease pathogenesis. To dissect the genetic architecture and proteomic regulation underlying MASLD, we generated a genetically diverse mouse cohort through a four-way cross of founder strains with varying susceptibility to liver disease, producing 444 F2 mice with a spectrum of phenotypes and genotypes. Utilizing deep proteomic profiling of the livers of this population, we identified quantitative trait loci (QTL) for 2,652 proteins, spanning over 4,000 unique genomic loci, and distinguished cis- and trans-acting regulatory mechanisms. Integrating proteomic, genomic, and phenotypic data reveals key regulatory loci and candidate proteins influencing disease progression, creating a mineable proteogenomic resource for MASLD research. We utilize this resource to identify the E3 ubiquitin ligase Ubr1 as a candidate central regulator connecting proteostasis and lipid metabolism, with genetic polymorphisms that may alter its abundance and predispose to metabolic dysfunction. This study demonstrates how high-throughput, deep proteome profiling integrated with QTL mapping can reveal complex gene-protein networks governing MASLD susceptibility and progression, offering novel insights for biomarker discovery and therapeutic targeting.

## Full Text Availability

The license terms selected by the author(s) for this preprint version do not permit archiving in PMC. The full text is available from the preprint server.

